# Redefining *postmortem* interval estimation: the need for evidence-based research to bridge science and justice

**DOI:** 10.3389/fmicb.2025.1646907

**Published:** 2025-10-17

**Authors:** Maria J. Teixeira, Daniel J. Barbosa, Ricardo Jorge Dinis-Oliveira, Ana R. Freitas

**Affiliations:** ^1^UCIBIO - Applied Molecular Biosciences Research Unit, Translational Toxicology Research Laboratory, University Institute of Health Sciences (1H-TOXRUN, IUCS-CESPU), Gandra, Portugal; ^2^Associate Laboratory i4HB - Institute for Health and Bioeconomy, University Institute of Health Sciences - CESPU, Gandra, Portugal; ^3^Department of Public Health and Forensic Sciences and Medical Education, Faculty of Medicine, University of Porto, Porto, Portugal; ^4^FOREN - Forensic Science Experts, Lisboa, Portugal; ^5^UCIBIO, Unidade de Ciências Biomoleculares Aplicadas, Faculdade de Farmácia, Universidade do Porto, Porto, Portugal; ^6^Laboratório Associado i4HB, Faculdade de Farmácia, Instituto para a Saúde e a Bioeconomia, Universidade do Porto, Porto, Portugal

**Keywords:** *postmortem* interval, thanatomicrobiome, thanatochemistry, thanatobiology, biomarkers, forensics sciences

## Abstract

Classical methods for *postmortem* interval (PMI) estimation have been applied for nearly a century. Contrary to the notion of being simple or easily accessible, these approaches require highly specialized training, including a medical degree, postgraduate specialization in forensic pathology, and extensive practical experience. Classical PMI estimation relies on observable physical and chemical changes in the human cadaver, such as *rigor mortis, livor mortis, algor mortis*, and transformative processes during decomposition. These methods are fundamental in medicolegal practice but remain largely influenced by environmental and individual variability. Recent advances in forensic research, particularly in microbiology and biochemistry, have introduced innovative approaches that complement traditional methods, offering greater accuracy and reliability, though resource-intensive. Emerging approaches leverage the predictable *postmortem* succession of microbial communities (thanatomicrobiome) and biochemical alterations in cadaver fluids and tissues. Techniques such as metagenomics, metatranscriptomics, and metabolomics enable detailed analysis of these changes, while computational models and machine learning further refine PMI estimates. Despite advancements, challenges persist, including variability due to environmental factors and limited access to human decomposition data. Integrating multi-omics approaches and artificial intelligence offers a path forward, addressing these limitations and enhancing the accuracy of PMI estimation. This review provides a comprehensive overview of PMI estimation, critically examining classical approaches and highlighting cutting-edge methodologies rooted in thanatomicrobiology and thanatochemistry. We emphasize the transformative potential of multi-omics integration and artificial intelligence in improving PMI accuracy. Importantly, we propose a paradigm shift: redefining PMI estimation through evidence-based, interdisciplinary research that bridges scientific rigor and judicial application. Transdisciplinary collaboration and standardized methodologies will be essential to translate emerging knowledge into robust forensic tools that serve both science and justice.

## 1 Introduction

Forensic sciences encompass a wide range of specialized disciplines, including forensic medicine, entomology, microbiology, toxicology, anthropology, geology, and ballistics, among others (Dinis-Oliveira and Magalhães, 2016). Each field follows rigorous, standardized procedures for the collection, preservation, and interpretation of evidence, and their integration is indispensable for unraveling complex cases and supporting judicial processes (Katz and Halámek, 2016; Saks and Koehler, 2005).

The *postmortem* interval (PMI), the time elapsed since death is a core responsibility in forensic medicine, yet it remains scientifically and procedurally challenging (Zapico and Adserias-Garriga, 2022). Contrary to some representations, classical PMI estimation methods, including the assessment of algor mortis, rigor mortis, and livor mortis, are neither simple nor readily accessible to all. The accurate application and interpretation of these techniques require highly specialized expertise in forensic pathology, as well as a thorough understanding of the multitude of variables affecting *postmortem* changes (Zapico and Adserias-Garriga, 2022; Iancu et al., 2016; Metcalf et al., 2013; Sutton and Byrd, 2020).

Contrary to the perception of being simple or easily accessible, classical PMI estimation methods, such as the evaluation of *algor mortis, rigor mortis, livor mortis*, and other transformative *postmortem* processes, are complex, highly specialized, and legally restricted. Accurate interpretation requires extensive postgraduate training in forensic pathology or legal medicine, often encompassing a decade or more of academic study and supervised practice. In many jurisdictions, only licensed medical doctors with such specialization are legally authorized to perform autopsies and apply ancillary PMI estimation methods, such as histology, entomology, radiology, or biochemical analysis. Furthermore, the routine implementation of these ancillary investigations is generally limited to accredited forensic institutions with the necessary infrastructure and financial resources, restricting their availability outside major medicolegal centers (Ruiz López and Partido Navadijo, 2025; Secco et al., 2025).

The decomposition of a human cadaver is a complex process driven by enzymatic autolysis, microbial proliferation, and environmental factors. Microorganisms, including bacteria, fungi, and protozoa, interact with conditions such as temperature, humidity, pH, and oxygen availability to shape the pace and nature of decay (Iancu et al., 2016; Cláudia-Ferreira et al., 2023; Vass, 2011). Temperature and humidity are major modulators, while pH and oxygen influence both microbial dynamics and *postmortem* chemical transformations (Vass, 2011).

Although many PMI estimation methods have been proposed, most remain impractical or highly environmentally sensitive, limiting their routine forensic application (Sampaio-Silva et al., 2013). Complementary disciplines such as microbiology, radiology, omics and botany have therefore gained importance (Zapico and Adserias-Garriga, 2022; Shrestha et al., 2023). In particular, the human microbiome follows predictable *postmortem* succession patterns, serving as a potential biological clock for PMI estimation (Hauther et al., 2015). In parallel, thanatochemistry—the study of *postmortem* biochemical changes—offers measurable indicators such as electrolyte shifts, protein degradation, and metabolite accumulation (Zapico and Adserias-Garriga, 2022; Bonicelli et al., 2022; Costa et al., 2015; Girlescu et al., 2021; Marrone et al., 2023). Combining microbial and biochemical approaches, supported by computational modeling and machine learning, provides a more robust and evidence-based framework for PMI estimation (Hauther et al., 2015; Meurs et al., 2019).

This review critically examines both classical and emerging PMI estimation methods, with a particular focus on the thanatomicrobiome and thanatochemistry, whose integration promises greater accuracy and reproducibility in forensic practice.

## 2 Methods

An exhaustive search was conducted using PubMed (U.S. National Library of Medicine) and Google Scholar, without restricting the search to a specific time period. The databases were queried with the following search terms: “thanatochemistry,” “thanatomicrobiology,” “biochemistry,” “forensics,” “*postmortem* interval,” “decomposition,” “microbiology,” “microbiome,” “microorganisms,” “forensic entomology,” “botany,” and “biomarkers.” Titles and abstracts of all articles available in English were meticulously reviewed, resulting in the selection of 112 articles.

Inclusion criteria required that publications be available in full text, written in English, and classified as either original research articles or reviews. Studies meeting these eligibility criteria were thoroughly analyzed, and relevant data were extracted for integration into our analysis.

## 3 Methods to estimate the *postmortem interval*

### 3.1 Classical vs. modern methods for PMI estimation

Classical methods have been in use for nearly a century. Classical PMI estimation relies on observable transformative processes (thanatomorphological phenomena) such as the progression of *algor mortis, livor mortis*, and *rigor mortis*, as well as putrefactive and decomposition changes. The onset and sequence of these processes are not only highly context-dependent, particularly influenced by environmental factors such as temperature and humidity, but also demand expert differentiation from *antemortem* and *perimortem* changes. These approaches are rooted in well-documented phenomena and can be applied without the need for advanced laboratory equipment. Simplicity, accessibility, and cost-effectiveness, since they do not require sophisticated tools, make them applicable in resource-limited settings. Although they have been studied and utilized for decades, they have proved to be highly ineffective. The persistence of these methods for more than a century reflects how forensic autopsy practice long resisted the incorporation of scientific and more accurate approaches. In other words, in several countries, PMI estimation follows almost the same procedures as the beginning of the twentieth century. While ancillary methods, including histology, entomology, and advanced biochemical analyses, play a central role in advancing the precision of PMI estimation, their implementation is not routine. These techniques require not only additional subspecialist expertise but also access to dedicated laboratory resources and significant financial investment. Moreover, due to strict legal and ethical guidelines governing the handling of human remains, such investigations are typically reserved for authorized forensic practitioners within institutional settings (Secco et al., 2025; Folkerth et al., 2025).

Over the last few years, modern biochemical and microbiological methods have emerged within research units as key tools for PMI estimation, though also with advantages and limitations. Modern approaches leverage advances in molecular biology, biochemistry, and microbiology, offering more accurate and reproducible PMI estimations. These include, for instance, the analysis of *postmortem* biochemical changes (e.g., degradation of metabolites or proteins) and shifts in microbial communities (thanatomicrobiome). By employing objective measurements, the subjectivity of the forensic expert is reduced. For example, proteomic analyses can identify protein degradation markers that correlate strongly with time since death. Despite modern methods may require high financial demands as it occurs with next-generation sequencing or mass spectrometry techniques, trained personnel have proven to be the major obstacle. Although for toxicology and genetics we have been developing experts in these fields, estimation of PMI will require proper education of the pathologist to incorporate these techniques in the casework routine. In the following sections, classical and modern methods are presented (Secco et al., 2025; Brockbals et al., 2023; Chhikara et al., 2025).

### 3.2 Methods based on early *postmortem* changes

**Figure 1:**
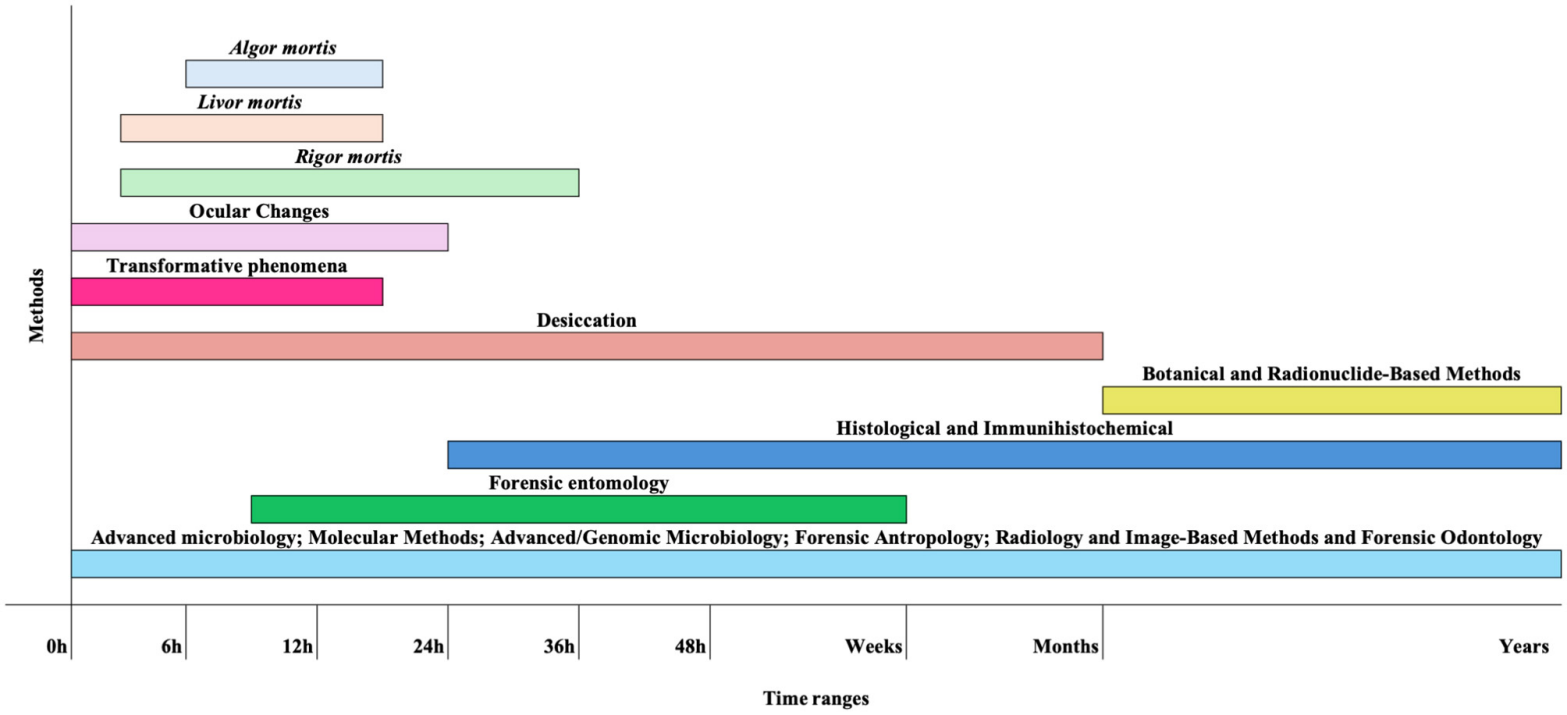
Schematic representation of key methods for estimating PMI and the approximate timeframes during which they can be applied. These timelines are only indicative, as decomposition is strongly influenced by multiple environmental and biological factors. For instance, transformative processes may occur within a few hours under hot and humid conditions, forensic entomology can provide estimates ranging from hours to weeks (excluding archaeoentomology), and radionuclide dating may still involve errors of several decades.

Over the years, various methods have been developed to estimate the PMI (Figure 1; Franceschetti et al., 2023), all having advantages and disadvantages (Table 1). Early *postmortem* PMI estimations typically rely on *algor mortis, livor mortis*, and *rigor mortis*.

**Table 1:** Overview of different methods used to estimate the *postmortem* interval, highlighting their key advantages and disadvantages.

**Methods**	**Advantages**	**Disadvantages**	**References**
*Algor mortis*	Simple and low-cost; useful in the first 24 h.	Cadaver temperature can be influenced by many factors (e.g., ambient temperature, clothing, and cadaver size).	Shrestha et al., 2023
*Rigor mortis*	Observable and quite predictable sequence. Offers the longest *postmortem* interval estimates.	Influenced by various factors, including ambient temperature and the individual's metabolic state at the time of death.	Kori, 2018; Madea, 2016; Amendt et al., 2010
*Livor mortis*	The progression of *livor mortis* occurs in distinct stages. Helpful for early PMI.	Only useful during the first few hours after death (becomes fixed around 8–12 h). Environmental factors (e.g., temperature) can affect its progression, making it an imprecise indicator of time of death.	Hauther et al., 2015
Ocular changes	Offers measurable changes (e.g., IOP, opacity) and a stable matrix for estimating the *postmortem* interval due to its resistance to contamination and environmental factors.	Requires careful interpretation due to environmental and individual variability.	Cláudia-Ferreira et al., 2023; De-Giorgio et al., 2021; Brooks, 2016
Histological and immunohistochemical	Changes in specific proteins/tissues provide valuable information. Useful in early PMIs.	Antigens such as insulin, thyroglobulin, or calcitonin allow only a rough PMI estimation, as many markers are effective only within limited timeframes, and results can be influenced by environmental factors and individual variability. Requires specialized techniques and expertise.	Metcalf et al., 2013; Kori, 2018; Mahalakshmi et al., 2016; Suhadi Pasaribu et al., 2024; Almulhim and Menezes, 2024
Desiccation	Visible progression. Helpful in advanced Decomposition.	Influenced by climate. Not suitable for precise PMI.	Brooks, 2016; Almulhim and Menezes, 2024
Forensic entomology	Useful in intermediate periods (days to weeks) using early colonizers. Well-documented insect succession.	Dependent on timely insect access to the remains. May be compromised by delays in oviposition.	Hauther et al., 2015
Transformative processes	Gross observation is possible in the field. Useful in late stages.	High variability. Environmental and individual differences reduce precision.	Zapico and Adserias-Garriga, 2022; Cláudia-Ferreira et al., 2023; Madea, 2023; Adserias-Garriga et al., 2017
Accumulated degree days (ADDs) method	Improves PMI estimates by integrating temperature into decomposition, allowing regional adaptation.	Its applicability may vary across different geographical regions and environmental conditions.	Franceschetti et al., 2021
Molecular methods	High specificity. Potential for tissue-specific models.	RNA/DNA degrade quickly. Sensitive to environmental factors.	Wenzlow et al., 2023
Botanical evidence	Uses seasonal plant indicators, growth analysis, and species identification to assess time of death and cadaver relocation. Useful in buried bodies and outdoor settings.	Effectiveness depends on proper collection and preservation. Growth rates are influenced by climate and species variation. Requires ecological expertise.	Cláudia-Ferreira et al., 2023; Miller Coyle et al., 2005; Ferri et al., 2009; Ermida et al., 2022
Forensic radiology	Visualizes internal changes like gas accumulation. Supports decomposition scoring.	Requires imaging technology. Factors like gas formation and low cadaver temperature can affect image quality.	Sapienza et al., 2020
Radionuclide-based methods	Radiocarbon in soft tissues reflects atmospheric levels. Estimates the year of death within ~3 years.	Requires preserved soft tissues. Costly and complex. Limited use for older remains. Results can be affected by environmental contamination.	Bulman and McLeod-Henning, 2012; Ubelaker, 2014
Forensic odontology	Teeth preserve well beyond 7–10 days. Dental exams help estimate age and identity.	Need for standardization, improved accuracy, and larger validation studies. Results can be slow.	Ugrappa and Jain, 2021; Bianchi et al., 2022
Forensic anthropology	Effective in skeletonized remains. Helps estimate age, ID, gender, and PMI.	Estimates age at death visually. Large error range for PMI. Not suitable for early PMI.	Cláudia-Ferreira et al., 2023; Villa and Lynnerup, 2014
Biochemical Markers	Predictable changes in markers like K^+^, Hx, and glucose. Stability of fluids (e.g., vitreous humor).	Accuracy declines over time. Affected by temperature and health status.	Salam et al., 2012
Proteomics	Protein degradation follows time-dependent trends. Robust in degraded samples.	Requires expensive equipment (e.g., mass spectrometry). Trained personnel needed.	Parker et al., 2016; Pittner et al., 2016; Prieto-Bonete et al., 2019
Volatile Organic Compounds	Decomposition-specific chemical fingerprints. Useful for PMI fingerprinting.	Requires gas analysis tools. Affected by storage/environment.	Xia et al., 2019; Madboly et al., 2021; Mazzatenta et al., 2024
Metagenomics	Predictable microbial succession. Useful in late PMI. Supports “microbial clock” models.	Influenced by external factors (humidity, oxygen). Human cadaver studies are limited.	Cláudia-Ferreira et al., 2023; Aguiar-Pulido et al., 2016
Metatranscriptomics	Reflects gene expression. Time-sensitive markers.	RNA instability. Requires advanced laboratory techniques.	Madea, 2023; Javan et al., 2020; Pesko et al., 2020
Metabolomics	Integrates metabolic fingerprints. Highly sensitive.	Complex interpretation. Influenced by multiple internal/external variables.	Aguiar-Pulido et al., 2016; Javan et al., 2020; Aljeaid, 2024

PMI, *postmortem* interval; IOP, Intraocular pressure; RNA, Ribonucleic acid; DNA, Deoxyribonucleic acid; ID, identity; K^+^, Potassium cation; Hx, Hypoxanthine.

#### 3.2.1 *Algor mortis*

*Algor mortis* is the *postmortem* cooling of the cadaver, reflecting the cessation of thermoregulation after death. Heat is lost via conduction, convection, radiation, and evaporation (Shrestha et al., 2023), with the cooling rate influenced by environmental conditions, body mass, clothing, and position (Kori, 2018; Madea, 2016).

While frequently cited in textbooks, accurate PMI estimation via *algor mortis* is complex and legally restricted to qualified forensic specialists. Interpretation requires measuring internal temperature (usually rectal or hepatic) and applying models such as the Henssge nomogram, which integrates body weight, clothing, and ambient temperature (Madea, 2023). Moreover, sudden changes in ambient temperature, such as *postmortem* refrigeration, can introduce significant error, and the method is not applicable in advanced decomposition stages (Bovenschen et al., 2021; Wardak and Cina, 2011). Recent developments include non-invasive thermometry and advanced numerical modeling, such as thermodynamic finite-difference algorithms and surrogate model-based parameter optimization, which allow individualized PMI estimation and can accommodate complex scenarios like variable ambient conditions and non-standard body postures (Wei et al., 2023). These approaches have demonstrated improved accuracy over traditional nomogram methods, with errors reduced to less than 1 h in controlled studies (Wilk et al., 2022).

#### 3.2.2 *Rigor mortis*

*Rigor mortis*, the *postmortem* stiffening of muscles, arises from adenosine-5′-triphosphate (ATP) depletion and actin-myosin binding until proteolytic enzymes degrade muscle fibers (Shrestha et al., 2023; Kori, 2018). It typically begins ~2 h after death, peaks at 6–8 h, persists for 12–24 h, and gradually resolves by ~36 h (Shrestha et al., 2023; Anders et al., 2013; Bate-Smith and Bendall, 1949). Although *rigor mortis* can provide indicative PMI estimates, its onset and duration are strongly influenced by temperature, metabolic state, and *perimortem* activity. Reliable interpretation requires formal training in forensic pathology, and in practice, it is used in conjunction with *algor* and *livor mortis* assessments by certified professionals (Kori, 2018; Madea, 2016; De-Giorgio et al., 2021; Nioi et al., 2021). Interpretation of *rigor mortis* is limited by its sensitivity to environmental and individual factors. Higher ambient temperatures accelerate the onset and resolution, while lower temperatures prolong the process. Muscle fiber type also influences timing, with red muscle developing rigor earlier than white muscle (Kobayashi et al., 2000, 2004). Physical exertion or convulsions before death can hasten ATP depletion and precipitate an earlier onset (Huang et al., 2010). These confounding variables mean that *rigor mortis* alone cannot provide precise PMI estimates and must be integrated with other *postmortem* findings, such as *algor mortis* and *livor mortis* (Ruiz López and Partido Navadijo, 2025).

#### 3.2.3 *Livor mortis*

*Livor mortis*, or *postmortem* hypostasis, is the purplish-blue discoloration of dependent skin caused by gravitational blood pooling in vessels and capillaries (Madea, 2023). It typically appears within 30 min to 2 h *postmortem*, becomes pronounced and coalescent by 6–12 h, and eventually fixes as hemolysis and hemoglobin diffusion occur (Shrestha et al., 2023; Hauther et al., 2015; Madea, 2016; Mahalakshmi et al., 2016). Forensic significance lies in pattern recognition and blanching response, which can indicate time since death and movement of the cadaver (Bockholdt et al., 2005). Objective methods such as spectrophotometric analysis have improved reliability, but interpretation still requires specialist training to account for confounding variables and to apply mathematical models for PMI estimation (De Donno et al., 2022). Legal restrictions mandate that only qualified forensic practitioners, typically forensic pathologists, may perform and interpret these analyses, as misinterpretation can have significant judicial consequences (Madea, 2016, 2023; Bockholdt et al., 2005).

#### 3.2.4 Ocular changes

Ocular *postmortem* changes, such as corneal opacity, variations in pupil diameter, blood vessel fragmentation, retinal discoloration, and intraocular pressure decline, evolve in a time-dependent manner and can assist in PMI estimation. Among these, corneal opacity and intraocular pressure are the most significant indicators: opacity typically becomes noticeable after ~8 h, and intraocular pressure decreases markedly within the first 12 h *postmortem* (Cláudia-Ferreira et al., 2023; Brooks, 2016; Nath et al., 2021). Additional findings include corneal thickening, transient pupil dilation and fixation, sometimes followed by constriction during *rigor mortis*, and the “cattle-truck” fragmentation of retinal vessels. Modern tools, such as optical coherence tomography, can visualize *postmortem* changes in retinal layers and reflectivity (De-Giorgio et al., 2021; Nioi et al., 2021). Interpretation of ocular changes is complex and environment-dependent, requiring consideration of temperature, humidity, and individual physiological variables. Consequently, PMI estimation using ocular signs is legally restricted to qualified forensic pathologists or legal medicine specialists, and often demands access to specialized ophthalmologic or forensic imaging facilities.

The vitreous humor is a particularly valuable matrix for *postmortem* biochemistry (Cláudia-Ferreira et al., 2023; Brooks, 2016). Located between the lens and retina, it is relatively protected from bacterial contamination and environmental factors, providing a more stable composition than blood or cerebrospinal fluid. Its predictable biochemical changes (see Section 4.2) make it a preferred sample for accredited forensic laboratories, though its collection and analysis remain limited to properly equipped biochemistry facilities. Moreover, collection requires a refined technique to avoid blood contamination, which may compromise the interpretation of results. Cloudy or blood-stained samples should be discarded, as they may lead to erroneous results (Lendoiro et al., 2012).

#### 3.2.5 Histological and immunohistochemical methods

Histological and immunohistochemical analyses provide valuable insights for estimating the PMI by detecting progressive tissue decay and time-dependent protein changes (Salerno et al., 2022). Histological studies document structural alterations such as vacuolation of oral mucosa within ~4 h and dermal fragmentation by ~24 h *postmortem* (Suhadi Pasaribu et al., 2024). Immunohistochemistry (IHC) allows the detection of specific antigens, including glucagon in pancreatic tissue for up to 6 days or collagen fibers via specialized staining (Surendhar et al., 2023). Other markers, such as insulin, thyroglobulin, and calcitonin, can support approximate PMI estimation in selected cases (Madea, 2023).

Recent advances, such as multi-marker panels and proteomic profiling, have improved early PMI estimation, particularly within the first 24 h (Salerno et al., 2022). However, environmental conditions, inter-individual variability, and tissue integrity significantly affect reliability, and many protein markers are useful only within narrow *postmortem* windows. Consequently, histology and IHC are considered supplementary tools rather than standalone methods for PMI determination (Cláudia-Ferreira et al., 2023; Brooks, 2016).

Importantly, the application of these microscopic and molecular techniques is legally restricted to accredited forensic laboratories and requires highly trained professionals, typically forensic anatomopathologists. Routine implementation also depends on specialized resources and infrastructure, limiting their availability outside major medicolegal or academic centers. Indeed, microscopic and molecular analyses, such as collagen degradation assessment, osteocyte quantification, and immunohistochemical staining, are highly sensitive to pre-analytical variables, environmental exposure, and taphonomic processes, which can confound results if not properly controlled and interpreted by subspecialists (Previderè et al., 2025).

#### 3.2.6 Desiccation

*Postmortem* desiccation produces progressive color and texture changes in mucous membranes and delicate skin surfaces, advancing most rapidly in moist areas like the eyes, lips, and genitalia (Brooks, 2016). A classic sign is “tache noir,” a black discoloration along the scleral equator caused by corneal drying. As tissues dehydrate, the skin and mucous membranes become dry and leathery, and in arid environments, this process may culminate in natural mummification within 2–4 weeks (Almulhim and Menezes, 2024).

Although desiccation patterns provide supportive information for PMI estimation, their interpretation is highly context-dependent, influenced by environmental conditions, body position, and exposure. Accurate forensic assessment of these changes is legally restricted to qualified specialists, typically forensic pathologists, and should be performed within accredited facilities as part of an integrated, multi-method evaluation. This context dependency and need for expertise are consistent with the broader challenges in PMI estimation in forensic anthropology, where visual and morphological skeletal assessments, taphonomic analysis, and contextual/archaeological indicators all require integration with environmental and case-specific data to improve reliability (Nau et al., 2025).

### 3.3 Methods based on late *postmortem* changes

In later *postmortem* periods, classical methods become more challenging to apply, making techniques like forensic entomology and molecular testing more relevant (Roy et al., 2021).

#### 3.3.1 Forensic entomology

Forensic entomology estimates the minimum *postmortem* interval (minPMI) by analyzing the arrival, colonization, and development of insects on human cadavers (Bambaradeniya et al., 2023; Harvey et al., 2016). Decomposing tissues provide both nutritional resources and oviposition sites, supporting the life cycles of various insect species. Early colonizing flies (Order Diptera) are often the first to arrive, and estimating the age of their oldest immature stages, combined with the likely time of oviposition, allows entomologists to determine how long the remains have been exposed (Harvey et al., 2016; Siva Prasad and Aneesh, 2022). Since insect development is temperature-dependent, accurate estimation also requires meteorological data for the relevant period.

Forensic entomology can inform criminal investigations by supporting PMI estimation, and, in some cases, the location or manner of death (Harvey et al., 2016; Siva Prasad and Aneesh, 2022). The applicability of entomological evidence spans from hours to several weeks *postmortem*, depending on species succession, environmental access, and whether archaeoentomological timelines are excluded. Despite its utility, entomological analysis is highly specialized and requires formal training and access to accredited laboratories capable of species identification and developmental staging. These analyses are therefore legally restricted to qualified forensic professionals, and their results are most reliable when integrated with other *postmortem* indicators such as *algor mortis, rigor mortis, livor mortis*, and biochemical findings (Hauther et al., 2015).

#### 3.3.2 Transformative processes induced by autolysis, putrefaction, and decomposition

The physical and chemical transformations of a human cadaver, such as skin discoloration, bloating, fluid release, and tissue breakdown, form the basis of classical PMI estimation (Adserias-Garriga et al., 2017). Human decomposition proceeds through distinct phases—fresh, bloating, decay, and drying—each marked by specific thanatomorphological phenomena and chemical changes (Zapico and Adserias-Garriga, 2022; Cláudia-Ferreira et al., 2023). Decomposition begins immediately with autolysis, the enzymatic self-digestion of tissues, which disrupts cellular compartments, alters ionic balances (↑K^+^, ↓Na^+^/Cl^−^), and lowers pH. Organs rich in enzymes, such as the pancreas, gastric mucosa, and adrenal medulla, degrade first. Within 24–72 h, putrefaction predominates: endogenous bacteria (notably *Clostridia* and *Proteus* spp.) spread through tissues, producing gases (H_2_S, NH_3_), bloating, discoloration, fluid leakage, and venous marbling. Amino acid, lipid, and carbohydrate catabolism generate biogenic amines, organic acids, and volatile compounds responsible for the characteristic odor of decomposition (Madea, 2023; Peng et al., 2020; Wenzlow et al., 2023).

Environmental and intrinsic factors, including temperature, humidity, oxygen availability, and body storage conditions, heavily influence decomposition rates. Plastic wrapping, water submersion, or sealed environments can significantly delay putrefaction, while arid conditions promote desiccation and eventual natural mummification. The dry phase culminates in skeletonization, whose timing varies widely from weeks to months depending on conditions (Javan et al., 2019).

Although these transformative processes provide critical forensic information, their interpretation is complex and context-dependent, requiring specialized training in forensic pathology or legal medicine. Accurate PMI estimation from decomposition changes is legally restricted in most jurisdictions to accredited professionals and is most reliable when integrated with other *postmortem* indicators, including entomology, biochemistry, and thanatomicrobiome analyses. Indeed, the medical literature consistently emphasizes that decomposition rates and patterns are influenced by a multitude of biological and environmental factors, including temperature, humidity, burial conditions, and individual characteristics, making accurate assessment highly challenging and necessitating expert knowledge for reliable interpretation. Moreover, these assessments have significant medicolegal implications and require adherence to strict protocols and ethical guidelines (Ruiz López and Partido Navadijo, 2025; Weisensee and Atwell, 2024).

##### 3.3.2.1 Accumulated degree days (ADDs) method

Megyesi et al. (2005) proposed estimating the PMI of decomposed human remains using accumulated degree days (ADDs), which account for the cumulative effect of ambient temperature on soft tissue decomposition. Recognizing that decomposition is a gradual, region-specific process, the method scores observable changes in three cadaver regions: the head and neck (including cervical vertebrae), trunk (thorax, pectoral girdle, abdomen, and pelvis), and limbs (hands and feet). By incorporating temperature-dependent progression rather than relying solely on elapsed time, this approach offers more refined PMI estimates. However, the applicability of ADD-based models is highly context-dependent, as decomposition rates vary with geography, climate, and environmental conditions (Franceschetti et al., 2021). Forensic interpretation of ADD scoring therefore requires specialized training and is legally restricted to qualified practitioners in accredited settings, often in combination with other *postmortem* indicators. Indeed, specialized forensic training is essential to correctly score decomposition, select appropriate models, and interpret confounding variables, as improper application can lead to substantial errors, especially when models are used outside their validated geographic or environmental context and it is needed strict adherence to protocols, chain of custody, and ethical standards (Forbes et al., 2019; Giles et al., 2020).

#### 3.3.3 Molecular methods

Molecular approaches for PMI estimation rely on the analysis of nucleic acid integrity and degradation, including deoxyribonucleic acid (DNA), messenger ribonucleic acid (mRNA), and long non-coding RNA (lncRNA; Cláudia-Ferreira et al., 2023; Brooks, 2016). Progressive nucleic acid breakdown occurs *postmortem* due to enzymatic activity, microbial invasion, and environmental conditions, with temperature and humidity strongly influencing degradation rates (Wenzlow et al., 2023). Several experimental studies illustrate the potential of these methods. For example, Peng et al. (2020) developed mathematical models based on the *postmortem* expression of hypoxia—and apoptosis—related mRNA markers, such as HAF, AIF, and HIF-2α. Similarly, lncRNAs in brain tissue have shown promise as early PMI markers in animal studies (Gao et al., 2024), while DNA degradation patterns in dental pulp are being explored with next-generation sequencing (NGS; Bianchi et al., 2024).

Emerging strategies aim to enhance reliability. These include combining RNA and DNA analysis to develop more reliable models, using ADDs instead of calendar days to better account for temperature variations, and investigating the potential of stable RNA types, such as certain lncRNAs, for more precise PMI determination (Gao et al., 2024; Bianchi et al., 2024). While molecular methods are promising and highly innovative, they remain experimental, tissue-specific, and resource-intensive, and their forensic application is currently limited to accredited laboratories with specialized expertise. Indeed, the tissue specificity arises because molecular degradation rates vary widely between tissues (e.g., muscle, liver, brain, and dental pulp), and are influenced by environmental factors, agonal events, and cause of death, making universal models unreliable and necessitating careful selection and interpretation of the biological matrix. Moreover, most studies focus on short PMIs and animal models, with limited human data and lack of standardized protocols, further complicating clinical translation (Secco et al., 2025; Cianci et al., 2024).

#### 3.3.4 Botanical evidence

Botanical evidence can provide contextual information about the environment surrounding human remains, although its application in PMI estimation is limited (Cláudia-Ferreira et al., 2023; Miller Coyle et al., 2005). Plant succession patterns, such as grasses colonizing disturbed soil before shrubs and eventually trees, may indicate time since burial site disturbance, while seasonal cues such as the state of leaves, flowers, or fruits, can offer approximate time-of-year indicators (Miller Coyle et al., 2005). *In situ* plant growth analysis can help estimate the minimum time a cadaver has been at a location, but maximum PMI cannot be determined reliably. Plant species may also provide geographical context, helping to identify whether remains were relocated. Modern methods such as DNA barcoding and microscopic algal analysis can support species identification and link remains to nearby environments, including aquatic sites (Ferri et al., 2009). The forensic utility of botanical evidence is highly dependent on expert collection, documentation, and preservation, while remaining sensitive to climate, species variability, and environmental conditions. As a result, forensic botany is underutilized, requiring specialized knowledge and integration with other forensic methods to be effective (Ermida et al., 2022).

#### 3.3.5 Forensic radiology

Forensic radiology applies imaging techniques such as X-ray, computed tomography (CT), and magnetic resonance imaging (MRI) to document *postmortem* changes (Madea, 2023). These methods can identify gas accumulation, tissue alterations, and other structural, or internal changes that occur after death. Interestingly, *postmortem* imaging can sometimes yield superior image quality due to the absence of motion artifacts. However, gas formation and low cadaver temperature may also affect image quality (Sapienza et al., 2020).

Recent research has investigated *postmortem* CT (PMCT) combined with machine learning models for PMI estimation. In an experimental rabbit model, PMCT data from multiple tissues, including the brain, eyes, myocardium, liver, kidneys, and muscles, were successfully used to train a stacking model for PMI prediction (Shen et al., 2024). Despite its promise, forensic radiology for PMI estimation is highly specialized, requiring advanced imaging infrastructure and expert interpretation, and remains restricted to accredited forensic centers. *Postmortem* imaging modalities—such as PMCT and MRI—demand not only technical proficiency in radiological acquisition and processing, but also deep understanding of *postmortem* anatomical and physiological changes, which differ substantially from clinical imaging findings. Moreover, dedicated *postmortem* imaging suites, specialized scanners, and advanced post-processing software are required.

#### 3.3.6 Radionuclide-based methods

Radionuclide-based methods, including the analysis of isotopes such as ^210^Pb, ^210^Po, and ^14^C, are particularly useful for aging skeletonized remains (Cláudia-Ferreira et al., 2023). These methods, such as radiocarbon (^14^C) dating, are primarily applicable for relatively old remains (several years to decades *postmortem*) and are currently limited by both their precision (often ± several years or decades) and the requirement for preserved soft tissues. Their practical applicability for more acute forensic timeframes is, at present, minimal (Madea, 2023; Bulman and McLeod-Henning, 2012; Ubelaker, 2014).

#### 3.3.7 Forensic odontology

Forensic odontology primarily supports identification and age estimation through the examination of dental tissues, particularly dental pulp, and can also contribute to PMI estimation under certain conditions (Cláudia-Ferreira et al., 2023; Ugrappa and Jain, 2021). Teeth provide natural protection from environmental factors, preserving tissues for early and late *postmortem* analysis.

Techniques include morphological and histopathological assessments of mineral density and microscopic alterations; molecular analyses of DNA and RNA, including by PCR and NGS. Recent advancements demonstrate the potential of NGS for PMI estimation, especially beyond 7–10 days *postmortem*, with specific DNA degradation patterns and mutations in dental pulp correlating with PMI intervals (Bianchi et al., 2024). Despite this promise, forensic odontology for PMI estimation is highly specialized, requiring accredited laboratories, expert interpretation, and further validation studies to improve accuracy and standardization (Bianchi et al., 2022).

#### 3.3.8 Forensic anthropology

Forensic anthropology applies the study of skeletal remains to medicolegal investigations, supporting identification, age and sex estimation, ancestry assessment, trauma analysis, and, in some cases, PMI estimation (Cláudia-Ferreira et al., 2023). Anthropological evaluations are particularly relevant in late PMI scenarios, where soft tissues have largely decomposed or skeletonization has occurred.

PMI estimation in anthropology is typically based on Weisensee and Atwell (2024):

1. Visual and morphological assessment of skeletal changes, including bone weathering, cortical exfoliation, and surface cracking;
2. Taphonomic analysis, examining how environmental factors (e.g., soil composition, moisture, pH, temperature, and vegetation) have affected bone preservation;
3. Contextual and archaeological indicators, such as soil staining, associated artifacts, and stratigraphy, which provide insight into deposition time.

Age-related skeletal changes can help estimate age at death, which is often necessary to contextualize PMI. However, precision is limited, and late PMIs usually require broad time ranges due to variability in environmental exposure, burial conditions, and scavenger activity (Villa and Lynnerup, 2014). Advanced methods, including histological bone analysis, stable isotope studies, and radiocarbon or luminescence dating, can complement traditional assessments but are resource-intensive and typically restricted to specialized laboratories (Schrag et al., 2014).

It is important to note that no single method provides a precise and secure determination of time since death (Table 1), especially for late PMIs. The most effective approach often involves combining multiple techniques and considering various factors that influence the decomposition rate.

## 4 Unveiling microbial and biochemical markers for PMI estimation

### 4.1 Thanatomicrobiome analysis: an emerging tool for PMI estimation

Before the emergence of the microbiome era in forensic science, forensic investigations primarily relied on traditional physical evidence, such as footprints, tool marks, fingerprints, and blood spatter. These methods were rooted in Locard's Exchange Principle, which postulates that physical evidence is inevitably left at nearly every crime scene. In the late nineteenth century, microbes were first employed as forensic evidence, but their use was narrowly focused on assessing the pathogenicity and lethality of microorganisms (Zhang et al., 2023). The field was constrained by the technological limitations of the time and a limited understanding of microbial communities, which restricted its broader application in forensic investigations. However, the advent of NGS and other advanced molecular techniques has significantly expanded the role of microbiology in forensic science, opening new avenues for investigation and analysis (Cláudia-Ferreira et al., 2023).

The study of *postmortem* microbial communities, known as the “thanatomicrobiome,” introduced in 2014 by Can et al. (2014), has emerged as a crucial area in forensic sciences, providing valuable tools for estimating the PMI (Zapico and Adserias-Garriga, 2022). After death, the breakdown of physical and immunological defenses allows microorganisms, predominantly from the gastrointestinal tract, to colonize less accessible areas. Bacteria such as those from the Bacillota (e.g., *Clostridium, Bacillus*, and *Peptoniphilus*) and Pseudomonadota phyla are prominent predictors of PMI, contributing to decomposition by breaking down complex tissues through enzymatic activity (Pechal et al., 2014). These microbial shifts, collectively referred to as the “microbial clock,” follow predictable patterns and serve as temporal indicators during decomposition (Metcalf et al., 2013). Microbial markers provide a unique advantage in PMI estimation, especially in later *postmortem* periods when traditional methods such as *rigor mortis* or *livor mortis* are no longer applicable.

The necrobiome, including internal (endonecrotic) and external (epinecrotic) microbial communities, is integral to understanding decomposition. Internal communities, or the thanatomicrobiome, are found in the blood, cadaver fluids, and internal organs, while external epinecrotic communities inhabit surfaces like the skin and orifices (Benbow et al., 2019). Although easier to sample, epinecrotic communities are more susceptible to environmental and biological factors, which can affect their reliability in forensic applications (Cláudia-Ferreira et al., 2023).

Research using advanced techniques such as 16S rRNA sequencing and shotgun metagenomics has highlighted the utility of microbial markers in PMI estimation. Predictable microbial shifts occur in internal organs, while the soil surrounding a cadaver undergoes changes influenced by leachates. The combination of microbial data from multiple sites, such as skin, caecum, and soil, with computational models has significantly improved PMI accuracy. For example, microbial taxa such as *Clostridium* and *Rhizobiales* have been identified as strong predictors of PMI, particularly during the active decay phase (Burcham et al., 2019; Javan et al., 2016a; Lutz et al., 2020; Zhou and Bian, 2018). Despite these advancements, challenges remain. Variability in microbial composition due to lifestyle, diet, environmental conditions, and sampling methods can lead to inconsistencies in findings. Environmental factors like humidity, oxygen, and temperature also influence microbial dynamics but are often underexplored in forensic models (Cláudia-Ferreira et al., 2023). While the influence of these variables on microbial community changes is not fully understood, different studies suggest that bacterial succession could help estimate PMI, as certain groups (mainly from Gammaproteobacteria, Lactobacillaceae, and Clostridiaceae) are strongly linked to decomposition (Hyde et al., 2017). Specific bacterial taxa play key roles in decomposition, and some, like those from Pseudomonadota and Bacillota, have been proposed as PMI biomarkers (Cláudia-Ferreira et al., 2023). Javan et al. (2016b) highlighted species from the Bacillota phylum (*Clostridium, Bacillus, Peptoniphilus, Blautia, Lactobacillus*) as potential biomarkers. Nevertheless, contamination during sample collection and limited access to human cadavers remain significant challenges. Moreover, conflicting results across studies continue to hinder the identification of reliable biomarkers, due to variations in experimental models, sampled cadaver sites, microbial communities, and decomposition conditions. Additionally, most PMI studies rely on animal models, complicating the translation of results to human decomposition scenarios (Cláudia-Ferreira et al., 2023).

The incredible advancements in microbiome analysis, including high-throughput sequencing and machine learning, such as Random Forest regression models, have enabled the rapid identification of microbial communities even in degraded samples (Metcalf et al., 2013). These tools have expanded the potential of microbial markers, with the microbial clock showing particular accuracy in the first 48 h after death (Cláudia-Ferreira et al., 2023). Moreover, microbial signatures persist in the soil long after decomposition, helping in locating clandestine graves. To further refine PMI estimation, future efforts should focus on standardizing protocols, expanding datasets with human cadavers from diverse environments, and incorporating environmental variables into predictive models. By addressing these challenges, the integration of microbial and computational approaches holds great promise for advancing forensic investigations.

### 4.2 Thanatochemistry and biochemical markers

Thanatochemistry, commonly referred to as “*postmortem* chemistry,” investigates the chemical changes that occur in various cadaver fluids and tissues after death, with a focus on decomposition and biochemical transformations, while accounting for various influencing factors (Zapico and Adserias-Garriga, 2022; Salam et al., 2012). This field plays a critical role in understanding *postmortem* biochemical shifts, offering valuable insights for estimating the PMI (Vieira et al., 2024). By analyzing a range of biochemical markers, thanatochemistry provides a more robust and potentially reliable approach to PMI estimation, helping to address the limitations of traditional methods (Bonicelli et al., 2022; Costa et al., 2015; Girlescu et al., 2021; Marrone et al., 2023). However, despite its promise, this approach may still lack precision in certain contexts (Donaldson and Lamont, 2013).

Death initiates profound biochemical changes across all cadaver tissues, driven by the cessation of oxygen circulation, disruption of enzymatic reactions, cellular breakdown, and the halt in anabolic metabolite production (Donaldson and Lamont, 2013). Various biochemical markers, including Na^+^, K^+^, Ca^2+^, Cl^−^, urea, creatinine, and glucose, are present in different biological fluids and play a critical role in estimating PMI (Palmiere and Mangin, 2015). Among these fluids, vitreous humor and synovial fluid are particularly significant due to their protection from external influences, such as temperature fluctuations and the effects of chemical and microbial agents, as they are encapsulated within specific anatomical structures (Vieira et al., 2024).

Vitreous humor analysis has emerged as a valuable tool in thanatochemistry due to its protected anatomical location and slower autolytic changes compared to other biological matrices (Risoluti et al., 2019). Several biochemical markers in vitreous humor exhibit predictable trends *postmortem*, helping in PMI estimation. K^+^ concentration is one of the most extensively studied parameters for PMI estimation, as K^+^ levels rise linearly after death due to cell membrane breakdown and diffusion from surrounding tissues. This predictable increase has enabled the development of formulas to estimate the PMI. However, the accuracy of the method decreases with extended PMIs, particularly beyond a few days, and can be influenced by factors such as temperature and individual baseline variations. In addition to K^+^, other biochemical markers in vitreous humor, such as Na^+^, Cl^−^, glucose, and hypoxanthine (Hx), also display predictable *postmortem* trends. Hx, in particular, rises significantly after death and serves as a complementary indicator, while Na^+^, Cl^−^, and glucose levels gradually decrease (Salam et al., 2012). Recent advancements have enhanced PMI prediction by integrating multi-parameter approaches, such as combining K^+^ and Hx measurements, and by analyzing other electrolytes like Ca^2+^. Advanced techniques, including spectroscopy, chemometrics, thermogravimetry, and Inductively Coupled Plasma Optical Emission Spectrometry (ICP-OES), have further refined data interpretation and improved model accuracy (Ding et al., 2017; McCleskey et al., 2016). Machine learning applications are also being explored to optimize these methods, providing more robust and precise PMI estimation tools (Risoluti et al., 2019).

Synovial fluid becomes especially valuable in PMI estimation when vitreous humor is unavailable, as the progression of its K^+^ concentration progression closely mirr|lors that of vitreous humor (Madea, 2005). Tumram et al. (2011) demonstrated that increasing K^+^ and decreasing glucose levels in synovial fluid are at least as accurate, if not more accurate, than those in vitreous humor for determining the PMI. More recently, K^+^ concentration in synovial fluid was proposed as the most promising biomarker to estimate PMI (Vieira et al., 2024). Furthermore, a study by Srettabunjong et al. (2019) revealed significant correlations between the concentrations of Na^+^, K^+^, glucose, lactate, urea, uric acid, and creatinine in both fluids (Table 2), although Cl^−^ and Mg^2+^ concentrations did not show the same level of correlation.

**Table 2:** Biochemical markers *postmortem* fluctuations.

**Biochemical markers**		**Levels after death**	**References**
Glucose	Vitreous humor	↓ gradually	Salam et al., 2012
	Synovial fluid	↓	Tumram et al., 2011
	Blood	↓ quickly	Costa et al., 2015
	Cerebrospinal fluid	↓ quickly	Umapathi et al., 2023
Potassium	Vitreous humor	↑ linearly	Salam et al., 2012
	Synovial fluid	↑ linearly	Tumram et al., 2011
	Cerebrospinal fluid	↑ significantly	Naumann, 1958; Peyron et al., 2019; Yadav et al., 2007
Sodium	Vitreous humor	↓ gradually	Salam et al., 2012
	Cerebrospinal fluid	↓	Naumann, 1958; Peyron et al., 2019; Yadav et al., 2007
Chlorine	Vitreous humor	↓ gradually	Salam et al., 2012
Lactate	Blood	↑ drastically	Costa et al., 2015
Creatinine	Synovial fluid	↑	Srettabunjong et al., 2020
	Blood	↑ sharply in the first 24 h and then stabilizes	Kikuchi et al., 2010
pH	Blood	↓ slightly during the first 5 days and then ↑ continuously	Kikuchi et al., 2010
Myoglobin	Blood	↑ in the blood, liver, and parotid glands	Miura et al., 2011; Nasir et al., 2024
C-reactive protein	Blood	↓ approximately 35%, but remain relatively stable for up to 6 days	Uhlin-Hansen, 2001
Chloride	Synovial fluid	↓	Tumram et al., 2011
	Cerebrospinal fluid	↓	Naumann, 1958; Peyron et al., 2019; Yadav et al., 2007
Calcium	Blood	↓ significantly and linearly with the putrefaction time	Costa et al., 2015
	Cerebrospinal fluid	Relatively stable	Naumann, 1958; Peyron et al., 2019; Yadav et al., 2007
Phosphate	Cerebrospinal fluid	↑ sharply	Naumann, 1958; Peyron et al., 2019; Yadav et al., 2007
Total protein	Cerebrospinal fluid	↑	Umapathi et al., 2023
Enzymatic activity	Cerebrospinal fluid	↑	Peyron et al., 2019; Spina-França et al., 1969
Hypoxanthine	Vitreous humor	↑ significantly	Salam et al., 2012
	Cerebrospinal fluid	Exponential ↑ observed during the early *postmortem* period	Peyron et al., 2019; Spina-França et al., 1969
Urea	Synovial fluid	↑	Srettabunjong et al., 2020
	Blood	↑	Costa et al., 2015
Uric Acid	Synovial fluid	↓	Srettabunjong et al., 2020
	Blood	↓ linearly	Costa et al., 2015
Total and Direct Bilirubin	Blood	↓ significantly and linearly with the putrefaction time	Costa et al., 2015
Transferrin	Blood	↓ significantly and linearly with the putrefaction time	Costa et al., 2015
Immunoglobulin M	Blood	↓	Costa et al., 2015
Creatine kinase	Blood	↑	Costa et al., 2015
Iron	Blood	↑	Costa et al., 2015
Adenosine Triphosphate	Muscle and organ tissues	↓ quickly in the brain, spleen, and kidney	Mao et al., 2013
Alkaline phosphatase	Muscle and organ tissues	↓ over the next 2 h in the dermal connective tissues	Oya and Asano, 1971
Acid phosphatase	Muscle and organ tissues	↓ over the next 2 h in the dermal connective tissues	Oya and Asano, 1971
Esterase enzymes	Muscle and organ tissues	↓ over the next 2 h in the dermal connective tissues ↑ in hair follicles and epidermis	Oya and Asano, 1971
Titin	Muscle and organ tissues	↓ quickly	Tuttle et al., 2014
Putrescine and cadaverine	Volatile organic compounds	Rapid ↑ 2 to 5 days in the muscle. Continuous ↑ from 6 to 10 days, but at a slower pace. ↑ in brain tissue.	Xia et al., 2019; Madboly et al., 2021
High mobility group box-1	Blood	Time-dependent ↑, peaking at day 3 at 14 °C before ↓ and plateauing; at 24 °C, it peaks at day 2	Kikuchi et al., 2010
Magnesium	Blood	may show a slight ↑	Naumann, 1958; Peyron et al., 2019; Yadav et al., 2007

Blood analysis can also provide valuable insights for estimating the PMI, as several biochemical parameters in blood undergo significant changes after death. Notably, total and direct bilirubin, urea (breakdown of amino acids and pyrimidine bases, with nitrogen being converted to urea primarily in the liver), uric acid, transferrin, and immunoglobulin M (IgM), along with enzymes such as creatine kinase (CK) and aspartate transaminase (AST), show linear correlations with PMI (Table 2). Additionally, electrolytes such as Ca^2+^ and iron also exhibit notable *postmortem* changes (Costa et al., 2015). Blood glucose concentrations decrease rapidly after death, while lactate levels increase, often reaching up to 60 times their *antemortem* values. This dramatic increase is attributed to the metabolic shift from aerobic to anaerobic pathways following death (Costa et al., 2015). Creatinine, a byproduct of phosphocreatine degradation, is also released into the bloodstream *postmortem*. Research indicates that creatinine concentrations rise sharply within the first 24 h after death, after which they stabilize. pH levels decrease slightly during the first 5 days and then continuously increase thereafter. High mobility group box-1 (HMGB1) has been proposed as a potential protein marker released upon necrosis as it shows a time-dependent increase, peaking at day 3 at 14 °C before decreasing and plateauing, while at 24 °C, it peaks at day 2 (Kikuchi et al., 2010). The oxidation-reduction potential (ORP), has a strong positive correlation with PMI across different temperatures (Yang et al., 2015). Myoglobin levels in blood, but also in liver and parotid glands, have been shown to correlate significantly with PMI: as the PMI increases, myoglobin concentration also rises, making it a reliable biochemical marker for estimating the PMI (Miura et al., 2011; Nasir et al., 2024). These biochemical alterations in blood can be used to create mathematical models to estimate PMI more accurately. However, environmental factors and individual variations can influence these changes. Thus, combining multiple biomarkers often yields the most reliable results. C-reactive protein (CRP) is a significant biochemical marker widely studied in forensic and clinical settings for its role in determining the cause of death and estimating survival time. *Postmortem* CRP analysis reveals distinct patterns that can provide valuable insights into the physiological state preceding death. Generally, CRP levels decrease by approximately 35% *postmortem* but remain relatively stable in blood for up to 6 days (Uhlin-Hansen, 2001). Elevated *postmortem* CRP levels are often indicative of an inflammatory process that was active prior to death. CRP levels also correlate with survival time, offering predictive value in various scenarios. In non-small cell lung cancer patients, higher CRP concentrations are associated with shorter survival times (median survival of 5.3 months compared to 18.5 months for patients with lower CRP levels; Xiao et al., 2019). Additionally, in acute deaths, CRP levels are generally lower in individuals who died immediately or within 6 h of the triggering event (Fujita et al., 2002). These findings underscore the potential of CRP as a reliable marker for estimating survival time and enhancing forensic investigations (Oh et al., 2016).

Cerebrospinal fluid (CSF) undergoes various biochemical and cellular changes after death, offering valuable insights for estimating the PMI and understanding the circumstances of death. Electrolyte levels in CSF shift noticeably *postmortem* (Table 2). While Na and chloride concentrations decrease, K^+^ levels rise significantly, showing an average increase of 1.21 meq/h up to 25 h *postmortem*. Calcium levels remain relatively stable, and magnesium may show a slight increase, although no strong correlation with PMI has been established. In contrast, phosphate levels increase markedly due to *postmortem* esterase activity (Naumann, 1958; Peyron et al., 2019; Yadav et al., 2007). Other biochemical markers in CSF, such as glucose and protein concentrations, change predictably after death. Glucose levels decline rapidly, while total protein levels increase, reflecting changes in protein profiles over time (Umapathi et al., 2023). Overall, the Na^+^-K^+^ ratio in CSF appears to be a particularly promising parameter for more accurate PMI estimation compared to individual ion values (Peyron et al., 2019).

Enzymatic activities, including AST and alanine transaminase (ALT), rise *postmortem*, alongside a pronounced exponential increase in Hx during the early *postmortem* period (Table 2; Peyron et al., 2019; Spina-França et al., 1969). Cellular changes in CSF also occur. Red blood cell counts increase as PMI progresses, while blood cells (pleocytosis) become more common, particularly in acute infections before death (Spina-França et al., 1969). While changes in CSF can offer valuable insights for PMI estimation, their reliability is influenced by numerous factors, including environmental conditions, injuries, individual characteristics, and the cause and timing of death. These variables can limit the accuracy of CSF-based methods, particularly for extended PMIs (Franceschetti et al., 2023).

Muscle and organ tissues undergo significant biochemical and structural changes after death, also offering valuable insights for estimating the PMI (Tuttle et al., 2014). Among the key biochemical markers, ATP and its degradation products provide a reliable framework for PMI estimation (Table 2). ATP levels in tissues decrease rapidly after death, following a predictable breakdown pattern: ATP → adenosine-5′-diphosphate (ADP) → adenosine-5′-monophosphate (AMP) → inosine-5′-monophosphate (IMP) → inosine (HxR) → Hx. This process occurs across multiple organs, including the brain, spleen, and kidney, and is temperature-dependent, with faster degradation at higher temperatures (Mao et al., 2013). Enzymatic activity also changes *postmortem* and varies by tissue type. In dermal connective tissues near wound edges, alkaline phosphatase, acid phosphatase, and esterase activities decrease within 2 h after death, while esterase activity in the epidermis and hair follicles may increase during the same period (Oya and Asano, 1971). These enzymatic changes, however, are influenced by environmental factors, which can complicate their use in PMI estimation (Buras, 2006).

Skeletal muscle tissue undergoes notable biochemical and structural alterations after death. While passive mechanical properties of muscle bundles and collagen content remain stable for up to seven days, the degradation of titin, a protein essential for muscle elasticity, decreases up to 80% over this period. Myosin heavy chain composition also remains unchanged within this timeframe (Tuttle et al., 2014). Histological examinations reveal no significant changes in muscle tissue at 3 h *postmortem*, but focal areas of autolysis appear by 24 h, with progressive autolysis occurring in subsequent days (Piegari et al., 2023).

Volatile organic compounds (VOCs) have emerged as valuable tools for estimating the PMI by analyzing the chemical changes that occur during decomposition. Among these compounds, putrescine and cadaverine stand out as particularly significant biomarkers. Different studies have explored their potential to provide reliable PMI estimates. Xia et al. (2019) analyzed VOCs in rat muscle samples collected at different PMIs, revealing a clear pattern of progression during decomposition. A study using rat muscle samples identified a three-stage decomposition model based on the number and total peak area of VOCs detected. In the first stage, spanning 1 day *postmortem*, no VOCs were observed. The second stage, covering days 2–5, showed a rapid increase in both VOC species and their total peak areas. During the final stage, from days 6 to 10, the increase in VOCs continued but at a slower rate (Xia et al., 2019).

Another study in brain tissue showed significant correlations of both putrescine and cadaverine with PMI (Madboly et al., 2021). Notably, putrescine proved to be a more accurate biomarker, with 99.5% of its variability linked to the timing of sampling, compared to 75.2% for cadaverine. This underscores the potential of these polyamines as precise indicators of decomposition stages. Using an electronic nose, Mazzatenta et al. (2024) recorded volatile metabolites emitted from human tissues and isolated muscle cells at various intervals (0, 24, 48, and 72 h *postmortem*). This approach enabled the creation of PMI volatomics fingerprints, highlighting critical decomposition points at 24 h for whole tissues and at 48 h for isolated cells.

All the biochemical changes aforementioned occur at different rates and can be influenced by various factors such as ambient temperature, cadaver composition, and the cause of death. Therefore, a combination of multiple biochemical markers is often used for more accurate PMI estimation. Ongoing research continues to identify and validate new biochemical markers for improved PMI determination (Mazzatenta et al., 2024).

## 5 Integrating biochemical and microbial signatures for enhanced PMI estimation

The integration of biochemical and microbial signatures of death presents a promising avenue for enhancing the accuracy of PMI estimation in forensic investigations. Traditional methods often yield significant margins of error, particularly beyond 48 h *postmortem*. Recent advancements in metabolomics, microbial analysis, and proteomics offer innovative approaches to refine PMI predictions. The integration of multiple approaches could yield the most reliable PMI estimations, balancing the strengths and weaknesses of each method.

Proteomics, the study of all expressed proteins and their changes under physiological and environmental conditions, is a versatile technique with numerous forensic applications due to its molecular-level insights (Duong et al., 2021). While DNA is often associated with biological evidence in forensic investigations, proteins are more robust and can endure in degraded samples, making them a valuable alternative or complement when DNA is damaged or unavailable (Parker et al., 2016). Protein degradation into smaller peptides, and ultimately, amino acids, is catalyzed by proteolytic enzymes (proteases), especially by the calpains, which are recognized to be responsible for a large part of proteolytic activity, as this enzyme class is ATP-independent in its activation (Madea, 2023). Recent evidence shows that *postmortem* degradation of certain proteins occurs in a predictable manner, making protein analysis a promising tool for PMI estimation (Pittner et al., 2016; Prieto-Bonete et al., 2019). Researchers propose examining the progression of protein degradation to correlate specific alterations with PMI ranges. This approach involves identifying suitable marker proteins, analyzing *postmortem* changes, and accounting for influencing factors. Degradation profiles can provide a relative timeline of events or, ideally, back-calculate to the time of death based on time-dependent curves. Proteomics directly detects biomolecular sequences, making it well-suited for analyzing poorly characterized evidence frequently encountered in forensic cases. For example, *postmortem* changes in bone protein profiles can provide insights into decomposition patterns, utilizing techniques like mass spectroscopy to link protein alterations to specific PMIs (Arora et al., 2024).

During decomposition, the evolving necrobiome produces various metabolites that can be biochemically detected. For instance, the *postmortem* increase in formic acid is likely due to bacterial putrefaction rather than anaerobic metabolism (Donaldson and Lamont, 2013). Tracking changes in microbial communities alongside metabolite concentrations could yield more accurate PMI estimates. Blood pH decreases rapidly *postmortem*, influencing microbial growth and succession patterns. Exploring the interplay between pH changes and microbial shifts can provide a more comprehensive framework for estimating the PMI (Donaldson and Lamont, 2013; Ewald, 2021). Microbial colonization occurs at organ-specific rates after death. For example, bacterial presence is detectable in the liver after 1 day, while 50% of pericardial fluid samples remain sterile even after 5 days (Cláudia-Ferreira et al., 2023; Ewald, 2021). *Postmortem* anaerobic metabolism leads to the accumulation of lactic acid and nicotinamide adenine dinucleotide reduced form (NADH), altering the biochemical environment and influencing microbial community composition (Donaldson and Lamont, 2013).

Metagenomics, metatranscriptomics, and metabolomics offer powerful approaches for analyzing microbial and biochemical activities during decomposition, providing valuable insights for PMI estimation. Metagenomics, the study of microbial community genomes, serves as the foundational step in microbiome research. Its primary goal is to infer the taxonomic profile of a microbial community. While whole-metagenome sequencing offers limited insights into functional profiles, metatranscriptomics—sequencing the community's entire transcriptome—provides a deeper understanding by identifying genes actively expressed under specific conditions. While metagenomics answers “what is the microbial community composition?”, metatranscriptomics explores “what genes are expressed together?”, and metabolomics focuses on “what byproducts are produced?” (Aguiar-Pulido et al., 2016). Metatranscriptomics can be associated with a thanatobiological approach for PMI estimation, which corresponds to a comprehensive gene expression analysis in the field of thanatobiology (Madea, 2023). This approach has shown marked changes in the expression of specific genes after death, and this change is independent of *postmortem* RNA degradation, enabling the identification of potential thanatotranscriptome biomarkers (Javan et al., 2020). Metabolomics offers a comprehensive view of biochemical changes during decomposition, with metabolite analysis revealing potential biomarkers for PMI estimation (Pesko et al., 2020). These changes can serve as biomarkers or “fingerprints” that can be complex to interpret due to variability influenced by external and internal factors. In this context, machine learning models have been applied to metabolomic data, uncovering hidden correlations and improving PMI prediction accuracy compared to traditional methods (Aljeaid, 2024).

Nevertheless, scarce studies have simultaneously assessed microbial changes along with biochemical analysis. Two remarkable studies by Liu et al. (2020, 2023) combined microbial community characterization, microbiome sequencing from different organs, and machine learning algorithms, while examining differences in the metabolism among different organs. In the first study, dating from 2020 (Liu et al., 2020), the authors described significant differences in the prevalence of particular bacterial species between the death point and advanced decay stages, suggesting *Enterococcus faecalis, Anaerosalibacter bizertensis*, and *Lactobacillus reuteri* as promising biomarkers in the decomposition process (Liu et al., 2020). Simultaneously, they reported that following a decrease after cadaver rupture on day 3, there was a marked increase in genes associated with carbohydrate and amino acid metabolism on days 7 and 15. In the other study, dating from 2023 (Liu et al., 2023), the authors demonstrated that during the first day of decomposition, genera such as *Agrobacterium, Prevotella, Bacillus*, and *Turicibacter* were identified as time-relevant in internal organs. Pathways linked to lipid, amino acid, carbohydrate, and terpenoid metabolism showed significant enrichment at 8 h compared to 0.5 and 4 h. These changes in microbiome composition and metabolic pathways were not only time-dependent but also organ-specific. Some amino acid metabolism pathways, such as those for glycine, serine, threonine, histidine, alanine, aspartate, glutamate, and lysine, were activated because of some bacterial behavior. For example, the lysine biosynthetic pathway is closely associated with bacterial activity, and two products of this pathway, lysine and meso-diaminopimelate (m-DAP), are directly involved in bacterial cell wall synthesis. After 12 h of decomposition, the pyruvate metabolism pathway was enriched in the kidney compared with the other organs. Lactic acid bacteria can produce pyruvate and lactate from carbohydrates, organic acids, and amino acids. These findings underscore the intricate relationship between microbial activity and metabolic pathways, highlighting their direct impact on biomarker fluctuations during *postmortem* decomposition. Another study by Hu et al. (2024) integrated microbial data with biochemical markers through a multimodal approach to improve the accuracy of PMI estimation. They highlighted the dynamic changes in microbial communities during decomposition, such as the shift from anaerobic to aerobic bacteria, and their potential as reliable PMI indicators. Additionally, biochemical markers like metabolites and VOCs offer complementary insights into the decomposition process. The authors advocated for a multimodal approach combining microbial and biochemical data, which provided more accurate and robust PMI estimation models when paired with machine learning. This integration represents a significant advancement in forensic sciences, offering objective tools for later-stage decomposition analysis. Zapico and Adserias-Garriga (2022) discussed new methodologies for PMI estimation by analyzing changes in human tissues and microbial communities, exploring the fields of thanatobiology and thanatomicrobiome.

Although studies integrating different research areas remain limited, the intricate interplay between metabolites, proteins, and microbes is undeniable. Protein degradation after death is systematically driven by microbial and enzymatic activity, which begins as metabolic processes cease (Zissler et al., 2021). Addressing these dynamic changes over time through multi-omics integration may provide a more comprehensive understanding of *postmortem* processes. By leveraging the combined strengths of metagenomics, metatranscriptomics, and metabolomics, researchers can uncover a detailed picture of the intricate relationship between microbial activity and biochemical changes during decomposition, enabling the development of more accurate and robust PMI estimation methods.

## 6 Conclusions and future perspectives

PMI estimation remains one of the most challenging tasks in forensic medicine. Contrary to the notion that traditional (classical) thanatochronological methods are “simple” or “easily accessible,” it is important to clarify that their use and interpretation require significant expertise and specialized training. In most jurisdictions, only highly qualified professionals, including medical doctors with advanced postgraduate specialization in forensic pathology or legal medicine, are legally permitted to examine human remains and perform related ancillary investigations. This extensive academic and practical preparation underscores the complexity and specialization necessary to estimate the PMI using classical approaches. Furthermore, ancillary methods such as histological analyses, forensic entomology, or radionuclide dating demand not only advanced technical skills but also substantial institutional and financial resources. Access to these methods is largely restricted to specialized laboratories and institutions, further highlighting the limitations of “routine” application in the forensic context.

Recent advances in molecular and microbial markers, including proteomics, metabolomics, microRNA, and mRNA degradation, as well as thanatomicrobiome succession, offer promising avenues for more precise PMI estimation. However, the literature reveals conflicting results regarding the reproducibility, accuracy, and generalizability of these methods (Secco et al., 2025; Wang et al., 2022). For example, studies on microRNA and mRNA stability demonstrate tissue-specific degradation patterns and significant influence from external factors, leading to variable results across different experimental models and populations. Similarly, microbial succession models, while conceptually robust, are affected by environmental predictors such as temperature, pH, and soil composition, resulting in high error rates and limited transferability between settings (Secco et al., 2025; Wang et al., 2022).

The integration of microbial and biochemical markers represents a promising frontier in PMI estimation, offering a multi-faceted approach to overcome the limitations of traditional methods. The thanatomicrobiome provides valuable insights into microbial succession during decomposition, while biochemical markers in fluids and tissues, such as protein degradation and VOCs, add critical temporal resolution. However, substantial gaps remain in standardizing methodologies, expanding geographically diverse datasets, and addressing the variability introduced by environmental and individual factors. Integrating microbial data with complementary methods like biochemistry analysis and metabolomics, and leveraging artificial intelligence, could revolutionize forensic microbiology, improving PMI estimation and other forensic applications.

Moreover, as recently proposed, integration of multi-omics strategies with artificial intelligence-based modeling has shown improved prediction accuracy in controlled studies, but these approaches require further validation in diverse forensic contexts and larger cohorts (Wang et al., 2022). The lack of standardized protocols, insufficient reporting of error rates, and limited cross-study comparability remain significant barriers to clinical translation.

To truly advance the field, future research must prioritize the development of unified protocols and robust, interoperable data frameworks. Collaborative efforts to harmonize methodologies and reporting standards will be essential to realize the full potential of these innovative approaches and to establish evidence-based guidelines for PMI estimation in forensic practice. This requires a redefinition of PMI estimation anchored in evidence-based and interdisciplinary science to better support the justice system. By bridging gaps between forensic science, computational biology, and legal standards, we can establish PMI estimation as a scientifically rigorous and judicially reliable tool in modern forensic practice. Finally, future research should also focus on multicenter validation of molecular and microbial markers, development of standardized analytical frameworks, and incorporation of environmental and individual-level predictors to enhance model robustness.
